# Supplementary material for: Phytochemical Profiling of Extracts from Rare Potentilla Species and Evaluation of Their Anticancer Potential
Source: Int J Mol Sci. 2023 Mar 2;24(5):4836. doi: 10.3390/ijms24054836 (PMC10002591; doi:10.3390/ijms24054836)
Supplement: Supplementary file 1 [file ijms-24-04836-s001.zip › ijms-2242463-supplementary.pdf]

International Journal of
Molecular Sciences

MDPI

Supplementary Material

Phytochemical Profiling of Extracts from Rare *Potentilla* Species and Evaluation of Their Anticancer Potential

Daniel Augustynowicz [1], Marta Kinga Lemieszek [2], Jakub Władysław Strawa [1], Adrian Wiater [3] and Michał Tomczyk [1,*]

[1] Department of Pharmacognosy, Faculty of Pharmacy with the Division of Laboratory Medicine, Medical University of Białystok, ul. Mickiewicza 2a, 15-230 Białystok, Poland; daniel.augustynowicz@sd.umb.edu.pl (D.A.), jakub.strawa@umb.edu.pl (J.W.S.),

[2] Department of Medical Biology, Institute of Rural Health, ul. Jaczewskiego 2, 20-090 Lublin, Poland; lemieszek.marta@imw.lublin.pl (M.K.L.)

[3] Department of Industrial and Environmental Microbiology, Institute of Biological Sciences, Maria Curie-Skłodowska University, ul. Akademicka 19, 20-033 Lublin, Poland; adrian.wiater@mail.umcs.pl (A.W.)

* Correspondence: michal.tomczyk@umb.edu.pl (M.T.), Tel.: 48-85-748-56-9

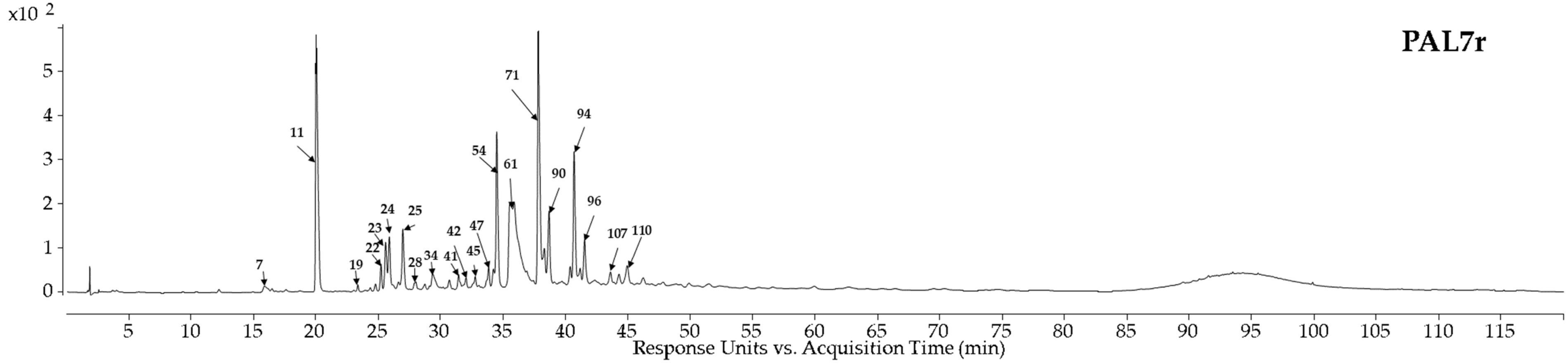

Supplementary Figure S1. The UV chromatogram with a designation of the main components of the analyzed **PAL7r** extract, recorded at length of 280 nm.

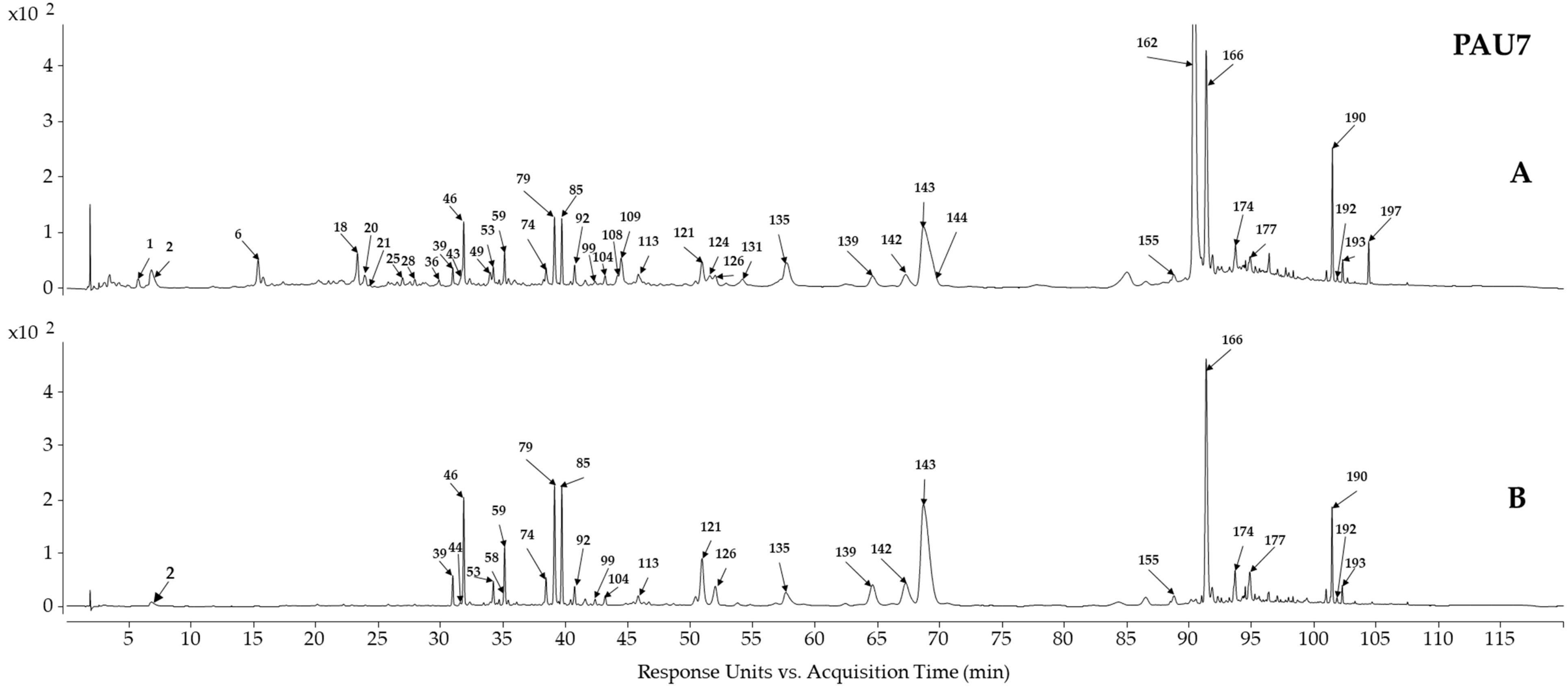

Supplementary Figure S2. The UV chromatograms with a designation of the main components of the analyzed **PAU7** extract, recorded at lengths of 280 nm (A) and 360 nm (B).

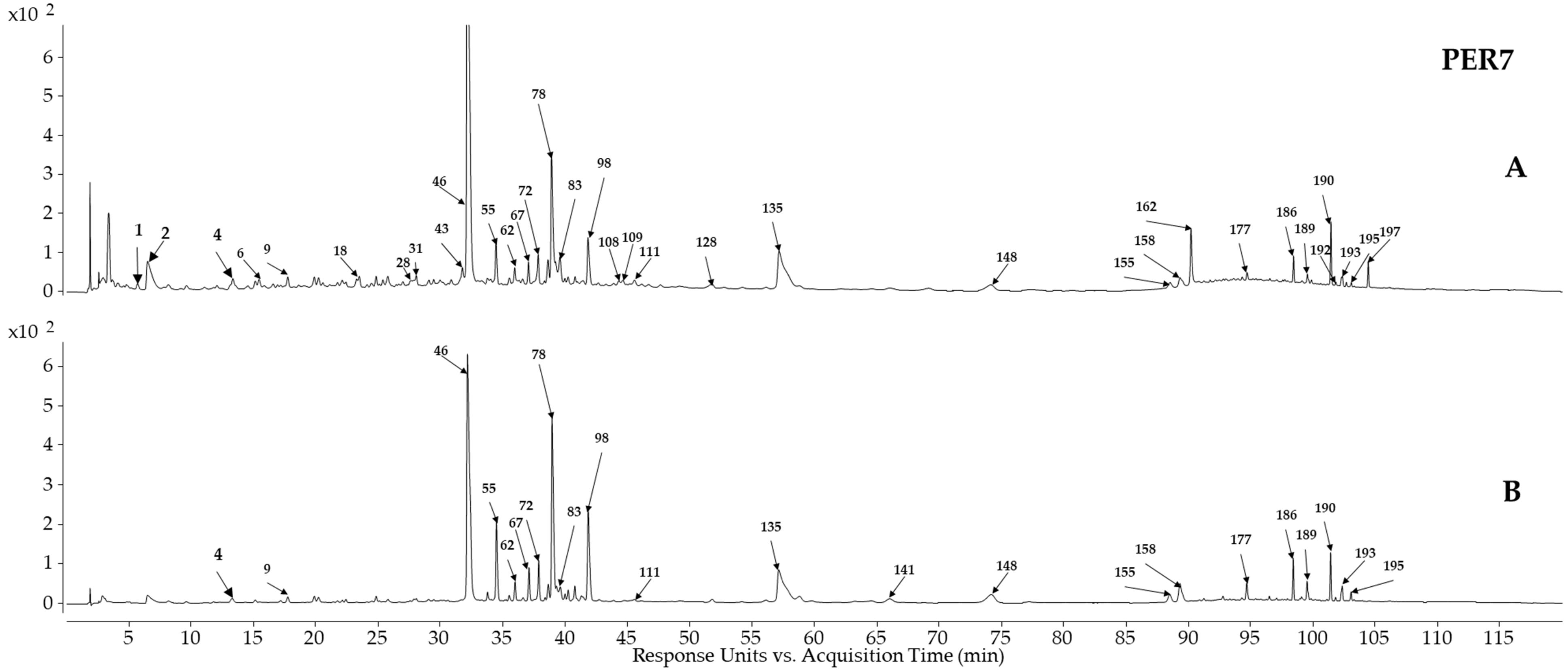

Supplementary Figure S3. The UV chromatograms with a designation of the main components of the analyzed **PER7** extract, recorded at lengths of 280 nm (A) and 360 nm (B).

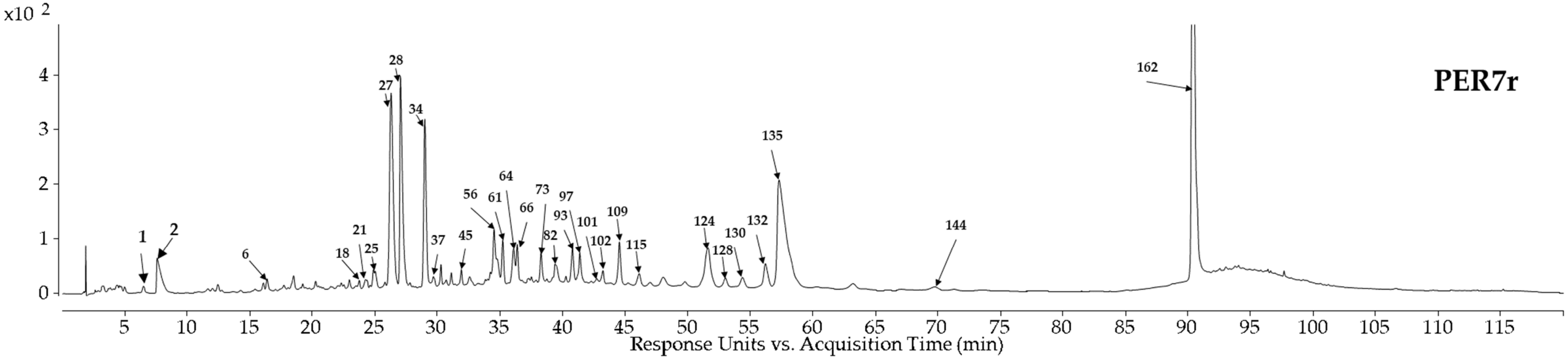

Supplementary Figure S4. The UV chromatogram with a designation of the main components of the analyzed **PER7r** extract, recorded at length of 280 nm.

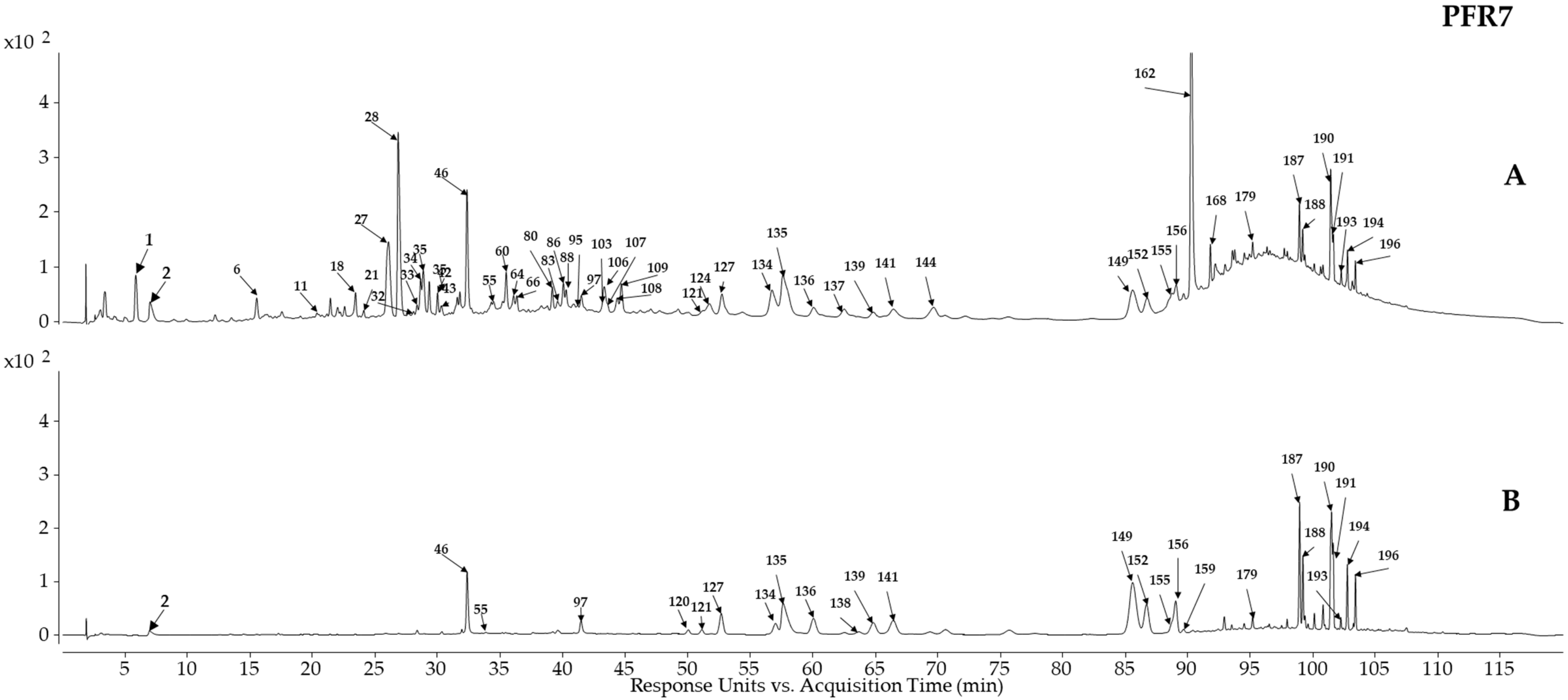

Supplementary Figure S5. The UV chromatograms with a designation of the main components of the analyzed **PFR7** extract, recorded at lengths of 280 nm (A) and 360 nm (B).

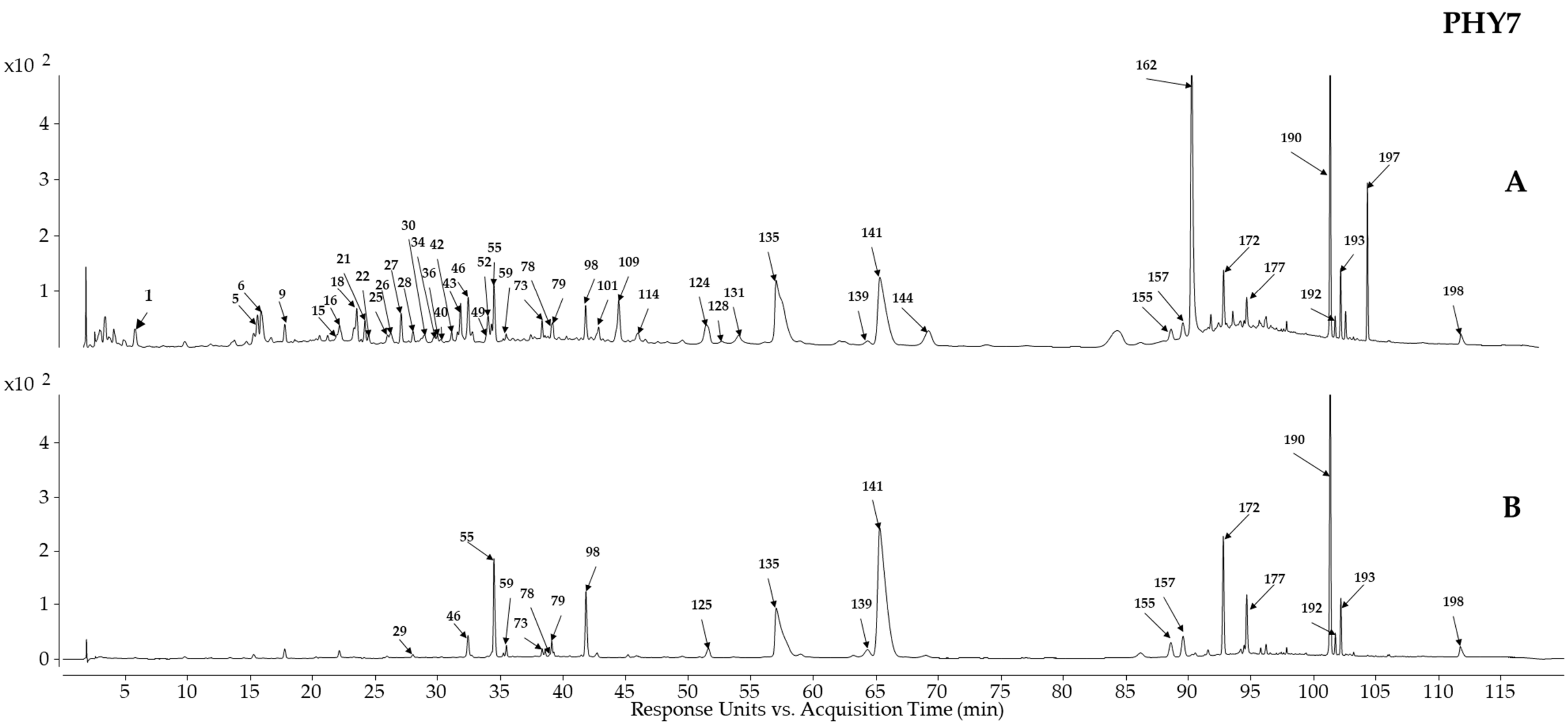

Supplementary Figure S6. The UV chromatograms with a designation of the main components of the analyzed **PHY7** extract, recorded at lengths of 280 nm (A) and 360 nm (B).

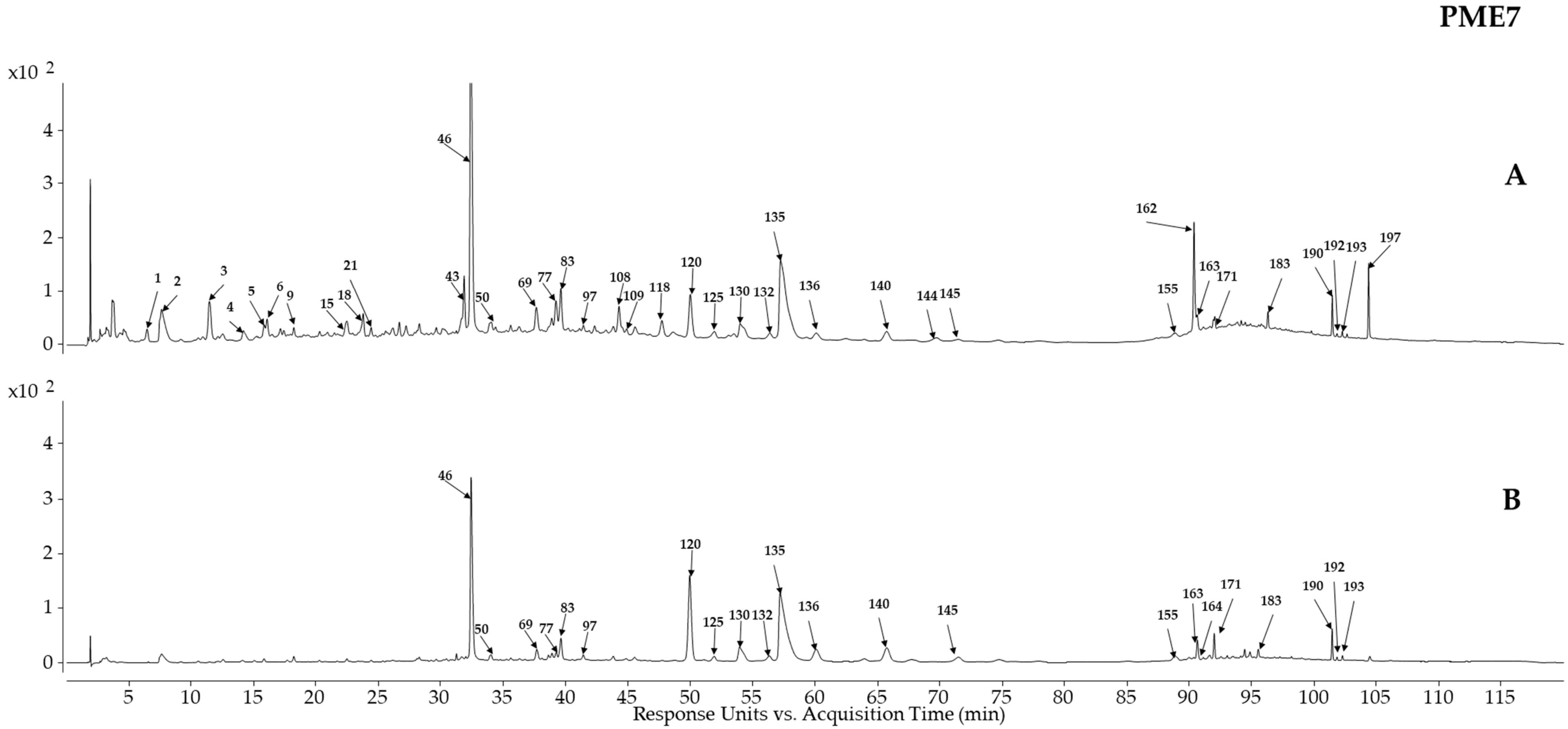

Supplementary Figure S7. The UV chromatograms with a designation of the main components of the analyzed **PME7** extract, recorded at lengths of 280 nm (A) and 360 nm (B).

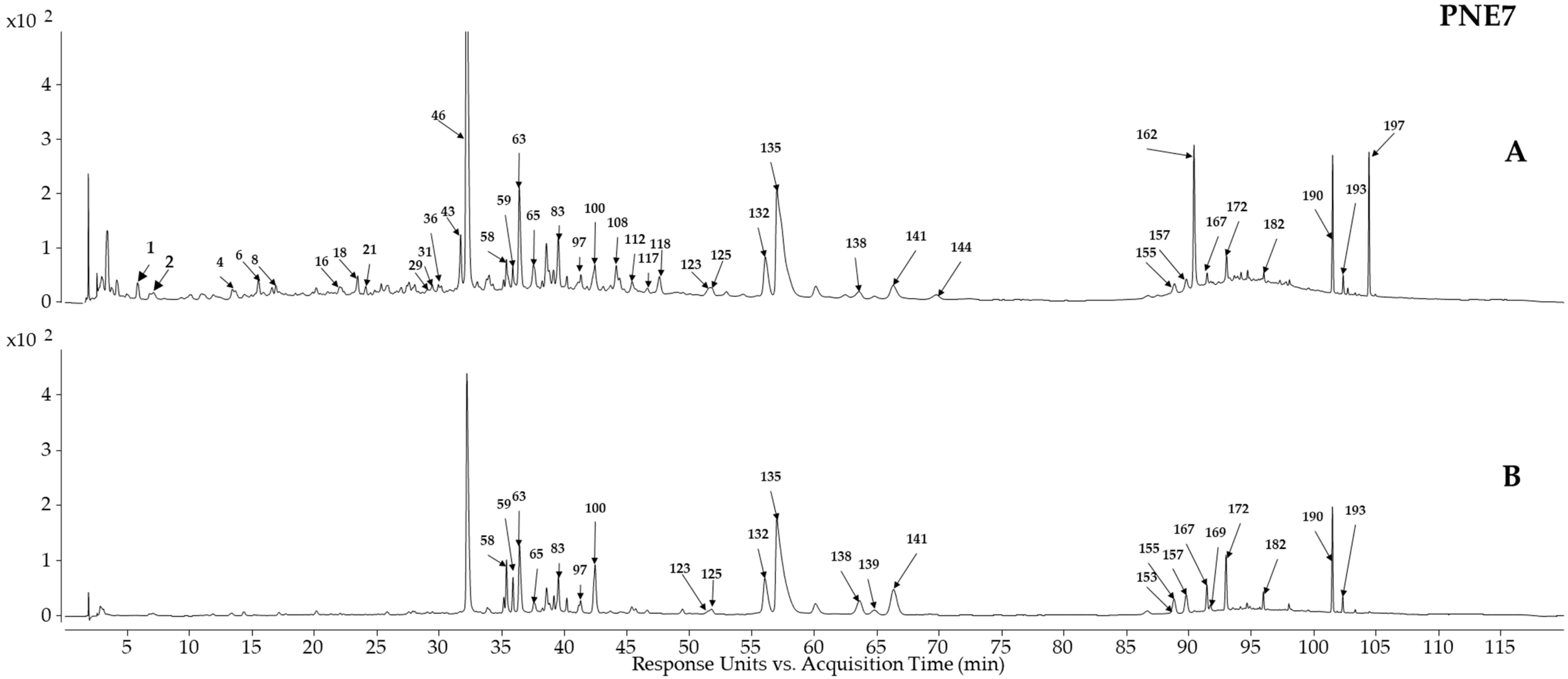

Supplementary Figure S8. The UV chromatograms with a designation of the main components of the analyzed **PNE7** extract, recorded at lengths of 280 nm (A) and 360 nm (B).

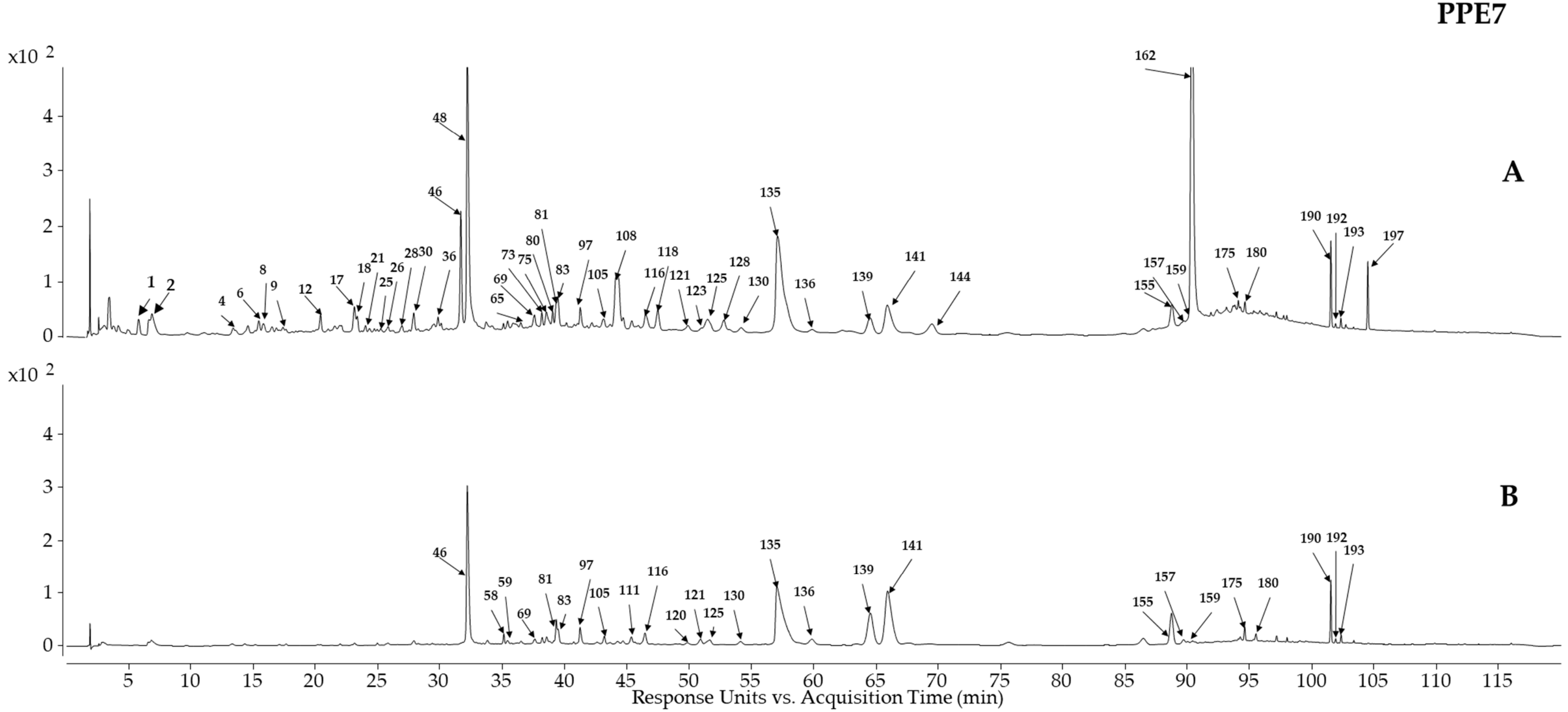

Supplementary Figure S9. The UV chromatograms with a designation of the main components of the analyzed **PPE7** extract, recorded at lengths of 280 nm (A) and 360 nm (B).

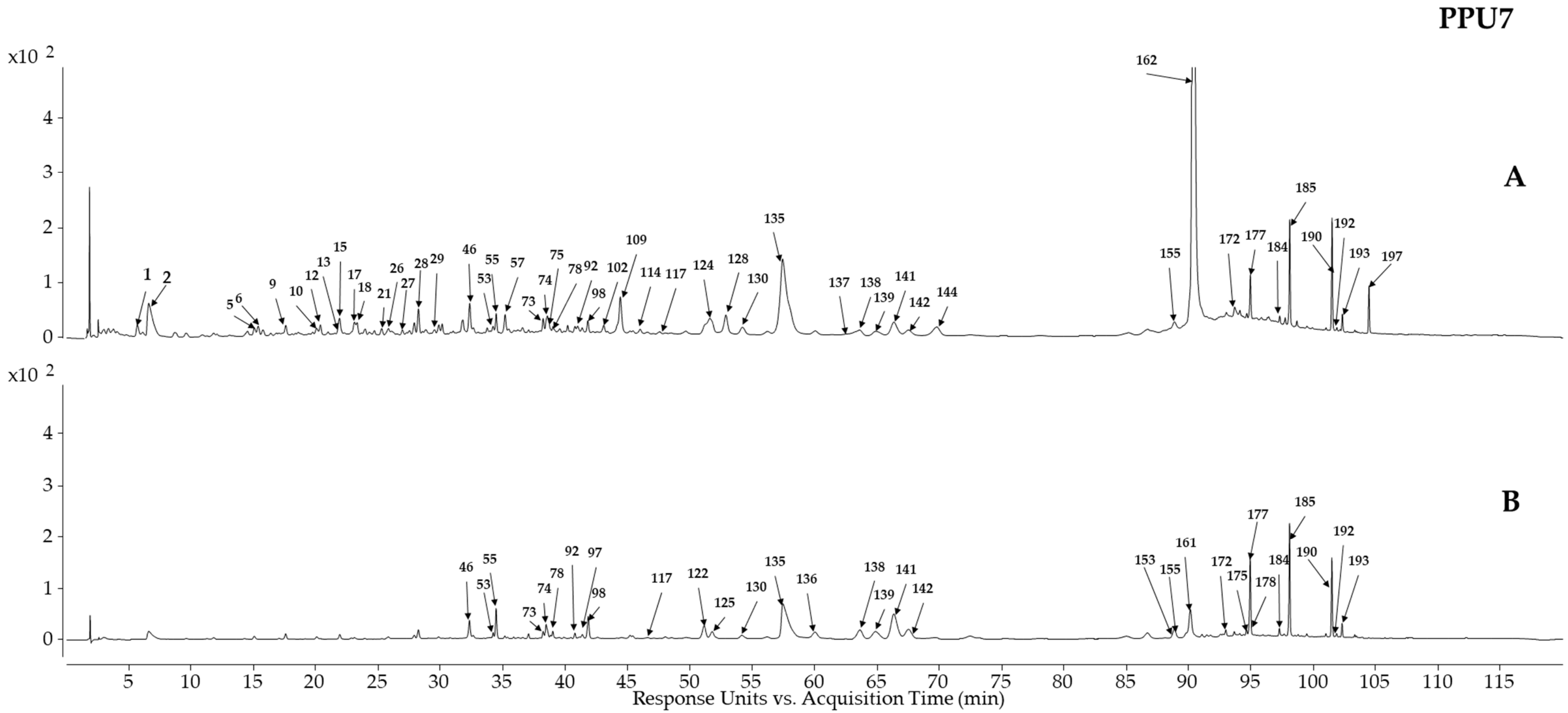

Supplementary Figure S10. The UV chromatograms with a designation of the main components of the analyzed **PPU7** extract, recorded at lengths of 280 nm (A) and 360 nm (B).

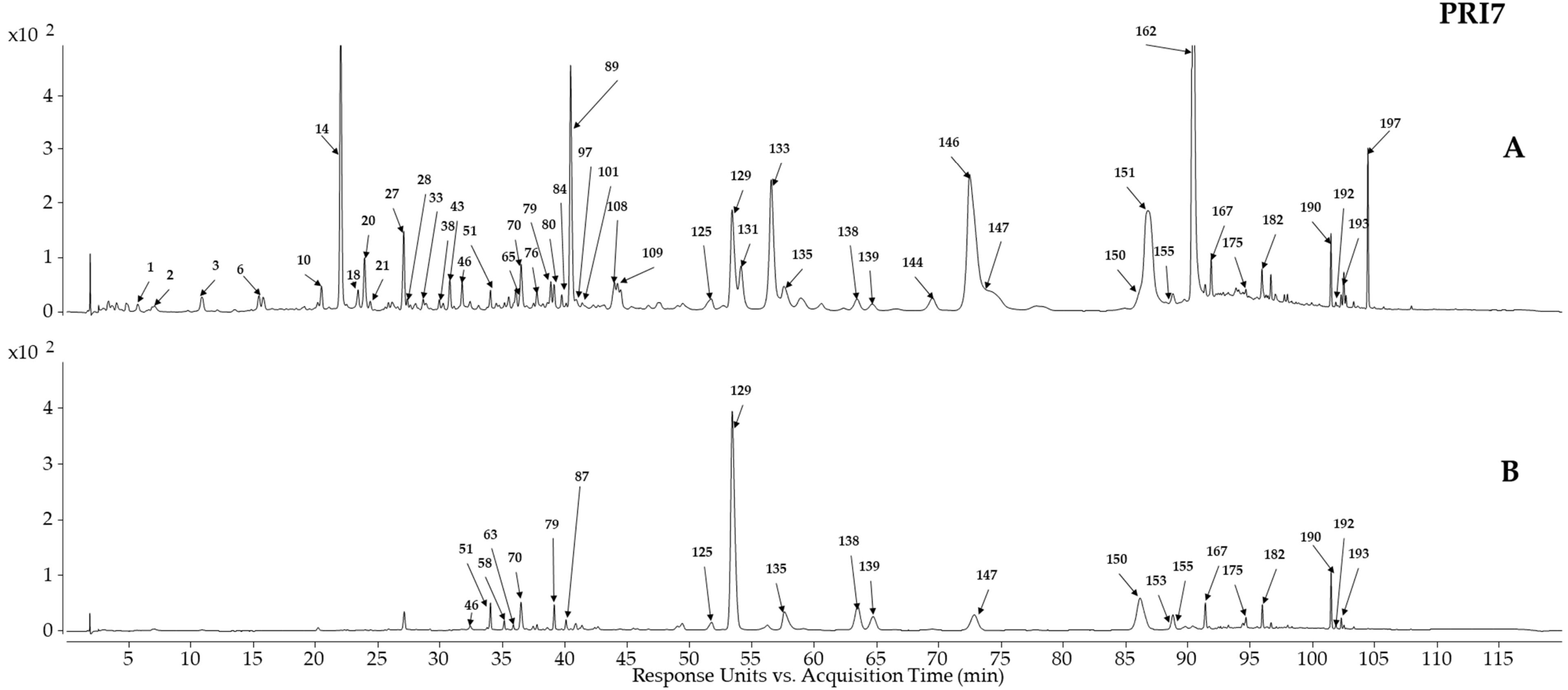

Supplementary Figure S11. The UV chromatograms with a designation of the main components of the analyzed **PRI7** extract, recorded at lengths of 280 nm (A) and 360 nm (B).

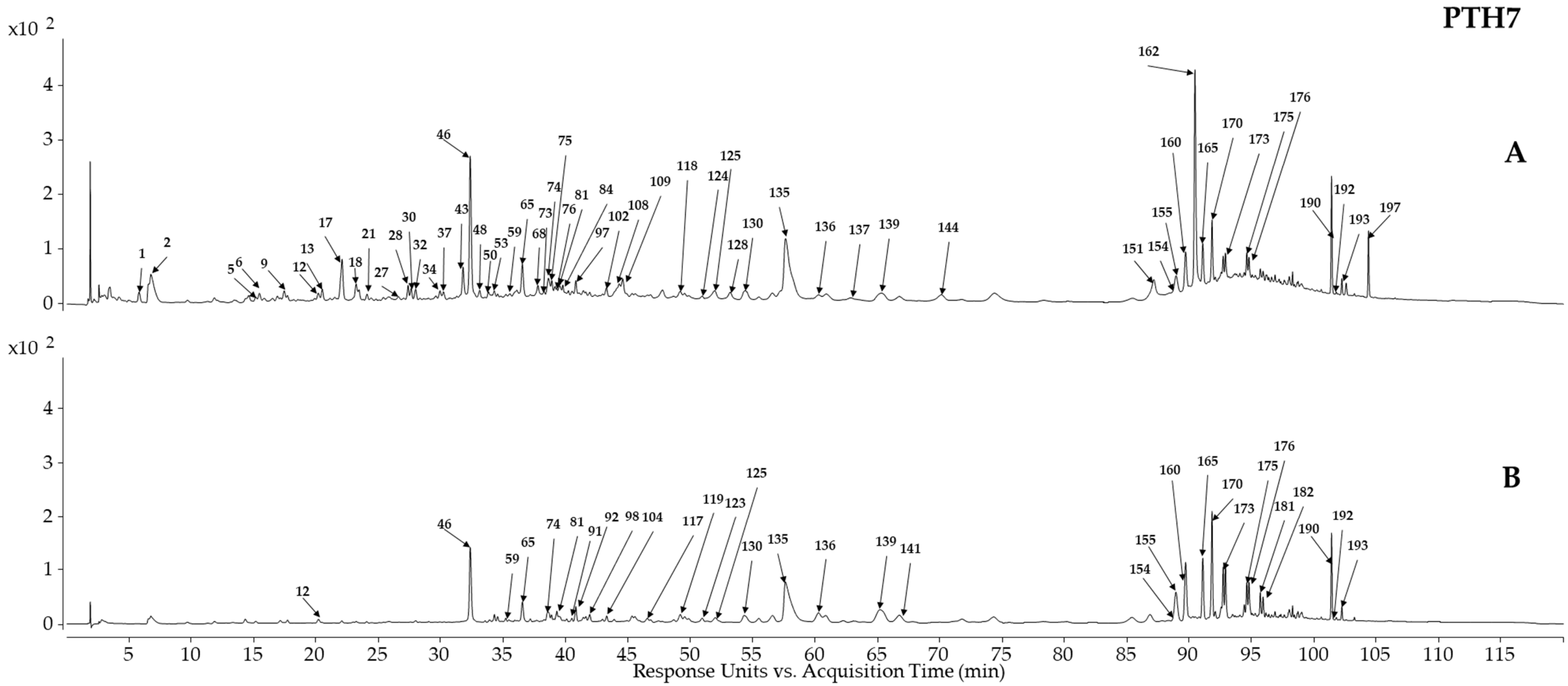

Supplementary Figure S12. The UV chromatograms with a designation of the main components of the analyzed **PTH7** extract, recorded at lengths of 280 nm (A) and 360 nm (B).
